# Investigation of biocompatibility and antibacterial properties of electrospun chitosan/ polyethylene oxide -based scaffolds containing propolis extract against *Enterococcus faecalis*, *Pseudomonas aeruginosa*, *Staphylococcus aureus* and *Staphylococcus epidermidis*

**DOI:** 10.1016/j.heliyon.2025.e42228

**Published:** 2025-01-23

**Authors:** Sayed Reza Ebrahimi, Mohammad Nikbakht, Milad Shahini Shams Abadi, Abolfazl Gholipour, Mitra Bagheri, Majid Validi

**Affiliations:** a Clinical Biochemistry Research Center, Basic Health Sciences Institute, Shahrekord University of Medical Sciences, Shahrekord, Iran; b Cellular and Molecular Research Center, Basic Health Sciences Institute, Shahrekord University of Medical Sciences, Shahrekord, Iran

**Keywords:** Chitosan, Propolis, Nanofibers, Antibacterial, Electrospinning

## Abstract

Bacterial infections represent a prevalent issue in healthcare settings. The indiscriminate use of antibiotics has contributed to the proliferation of antibiotic-resistant bacteria and complicating the treatment of such infections. Based on this, there arises a pressing need for alternative drugs to accelerate the treatment of a diverse array of Gram-negative and Gram-positive bacteria without inducing adverse side effects. In response to this need, we have developed chitosan (CS)/polyethylene oxide (PEO) nanofibers loaded with propolis extract using the electrospinning technique. We conducted an assessment of the antibacterial and antibiofilm activities of Chitosan/PEO nanofibers with varying concentrations of propolis against both Gram-positive and Gram-negative bacterial strains. The nanofibers demonstrated significant antibacterial activity against *Staphylococcus aureus*, *Staphylococcus epidermidis*, *Enterococcus faecalis*, and *Pseudomonas aeruginosa*. Additionally, according to the results obtained from the Congo red biofilm test, the optimal concentration of propolis in the nanofibrous mats exhibited a substantial antibiofilm effect against *S. aureus*, *S. epidermidis*, and *E. faecalis*. However, *P.aeruginosa* displayed growth and formed a relatively weak biofilm. The nanofiber scaffolds also demonstrated moderate antibiofilm activity against *S. aureus*, *S. epidermidis*, and *E. faecalis*, while displaying a weaker antibiofilm effect on *Pseudomonas aeruginosa* in the Tissue Culture Plate test. In summary, the biocompatible and antibacterial propolis extract-loaded Chitosan/PEO nanofibrous mats exhibit promising potential as effective wound dressings.

## 1 Introduction

The issue of antibiotic resistance is a worldwide concern. Despite numerous attempts, endeavors to combat antibiotic resistance in bacteria have proven ineffective thus far. Following the discovery of penicillin, there was optimism about eradicating resistant bacteria. However, this optimism was short-lived as penicillin rapidly lost its efficacy against a majority of susceptible bacteria. Furthermore, the widespread nature of antibiotic resistance is underscored by occurrences of oxacillin resistance in staphylococci, penicillin resistance in streptococci, vancomycin resistance in enterococci (and staphylococci), resistance in broad-spectrum Enterobacteriaceae, and carbapenem resistance in *P.aeruginosa* [1]. Easy access to antibiotics has fueled their indiscriminate and irrational usage, contributing to the emergence of novel resistant bacterial strains that often surpass the lethality of their predecessors. Consequently, this phenomenon has precipitated numerous widespread health-related problems [2]. The swift genetic mutations exhibited by these resistant bacteria render common antibiotics ineffective within a span of merely five years. Such profound levels of drug resistance compel scientific and medical professionals to explore alternative treatment modalities or to accelerate the development of novel drugs to combat these resilient bacterial strains [3]. Presently, researchers are heavily invested in exploring herbal medicines and natural compounds, aiming to uncover novel antimicrobial molecules capable of effectively treating a broad spectrum of Gram-negative and Gram-positive bacteria, while minimizing adverse side effects. Consequently, there is a growing trend towards utilizing natural antibacterial compounds, such as plant extracts, which offer promising alternatives to conventional antibiotics known for their limited efficacy and significant side effects [4]. Propolis, a natural substance, is created by bees using the resinous substance found in the buds of poplar and cone-bearing trees [5,6]. Propolis and its extracts as a “natural antibiotic”, are so effective in protecting, reinforcing, and repairing the hives. And it acts as an antifungal, antibacterial and antiviral barrier for hives [7]. The studies have identified more than 300 compounds such as resin (50%–70 %), oil and wax (30%–50 %), pollen (5%–10 %), amino acids, minerals, sugars, vitamins B, C and E, flavonoids, phenol, in the structure of raw propolis [8]. Additionally, propolis extract contains phenylpropanoids, cinnamic acids and their esters, glycerides, and caffeic acid phenethyl ester [9]. These compounds have positioned propolis as possessing antibacterial [10], antifungal [11], antiviral [12], anti-inflammatory, antioxidant, anticancer, and immunomodulatory [13] and wound healing [14]. Ideal antimicrobial dressings are the barrier to care for sores, that provide of a moist environment to healing and has antimicrobial effects against antibiotic-resistant bacteria. Also, they can remove exudate, increase debridement, and keep adequate water for wound healing [15]. Recently, wound dressings made by electrospinning method have received much attention due to their high porosity. In this technique, nano/microfibers are fabricated with dimensions in the nanoscale range, rendering them structurally similar to extracellular matrix proteins [[16], [17], [18]]. Features such as great porosity, wide surface area, nanoscale diameters, and teeny pore size make nano/microfibers noteworthy for biomedical usages. These properties intensify cellular respiration, regenerate the skin, retain humidity, remove secretions, and homeostasis. Biodegradable electrospun polymers create comfortable conditions for patients by reducing the need to change dressings. Moreover, the electrospinning technique enables the impregnation of nanofibers with antibacterial and therapeutic agents [19]. Chitosan is a positively charged biopolymer obtained through the deacetylation of chitin, which is found in the shells of marine animals and fungi [20]. Chitosan is distinguished by the presence of amine groups within its molecular structure. Properties such as biocompatibility, biodegradability, cell transplantation ability, and antibacterial activity, allows it applied in the medicine applications [21,22]. Due to its high viscosity, chitosan is challenging to process into nanofibers using the electrospinning method. Therefore, it is often mixed with other polymers to reduce its viscosity and facilitate the electrostatic spinning process. One of the polymers commonly used in combination with chitosan is polyethylene oxide (PEO), a non-toxic and water-soluble polymer. This synthetic polymer is known for its stability in acidic media. PEO, as a biocompatible polymer, can be electrospun into nanofibers, and enhancing the process of electrospinning chitosan [23]. Similar to the methodology in Singh et al.'s study, PEO was employed to alleviate the entanglement of chitosan chains and promote the production of nanofibers characterized by larger diameters and fewer bead formations [24]. The primary objective of this study was to fabricate wound dressings based on chitosan/PEO with enhanced antibacterial properties using the electrospinning method. To achieve this, propolis extract was incorporated to augment the antibacterial efficacy of the nanofibers. Subsequently, the morphology, cytotoxicity, antibacterial, and antibiofilm activity of the nanofibrous mats were evaluated.

## 2 Materials and methods

### 2.1 Materials

The propolis gum was sourced from Dolab village, Kurdistan province, Iran. Chitosan (CS) (MW: 20,000 g/mol) and Poly (ethylene oxide) (PEO) (MW: 80,000 g/mol)) were obtained from the Sigma–Aldrich (USA) brand. Gram-positive and Gram-negative bacterial strains were acquired from the Royan Institute (Tehran). Fetal Bovine Serum (FBS) and MTT (3-[4,5-dimethylthiazol-2-yl]-2,5 diphenyl tetrazolium bromide) were procured from Sigma-Aldrich. Other chemicals such as ethanol and acetic acid were available in the research institute.

### 2.2 Propolis extraction

The propolis was collected from honeybee colonies in Dolab village, Sanandaj city, Kurdistan Province, Iran. The extraction process involved immersing 40g of propolis in 200 ml of 70 % ethanol for a duration of 7 days. Afterward, the suspension underwent filtration using Whatman filter papers and was subsequently dried using a rotary evaporator. The resulting dried extract was gathered and stored at −20 °C for subsequent analysis.

### 2.3 The preparation process of CS/PEO and propolis-CS/PEO nanofibers

The process of creating CS/PEO nanofiber scaffold with and without propolis underwent several trial and error to achieve optimal results. In summary, to prepare the Chitosan solution, 0.3 g of chitosan was added to 10 mL of 1 % aqueous acetic acid under continuous mechanical stirring for 12 h. Simultaneously, Poly (ethylene oxide) (PEO) was fabricated by dissolving 0.03 g of the polymer in 1 ml of 0.5 M acetic acid with constant mechanical stirring for 2 h. Following the preparation of individual solutions of chitosan and PEO, a composite solution of CS/PEO was formed by mixing the obtained solutions at a ratio of 9:1. The mixture was subjected to continuous stirring using a mechanical stirrer for a duration of 45 min to formed a homogeneous composite solution. Propolis-CS/PEO solutions were then prepared by integrating various concentrations of propolis (ranging from 1 to 20 wt%) into the CS/PEO solution. Electrospinning was conducted under ambient conditions, utilizing specific parameters: A high voltage of 21 kV was applied to the polymer solution using an electrospinning apparatus (Nano azma, Iran). The distance between the tip and the collector was determined to be 10 cm. The injection speed of the solution into the electrospinning apparatus was also set to 0.5 ml/h to ensure a consistent supply of the polymer solution during the electrospinning process. The resulting electrospun fibers were deposited onto an aluminum sheet affixed to the surface of a grounded iron drum. Finally, the nanofibrous mat underwent vacuum drying process at 25 °C for 24h.

### 2.4 Characterization of nanofiber scaffolds

#### 2.4.1 Scanning electron microscopy of the nanofiber scaffolds

The morphology of the nanofibers (NFs) was examined using a scanning electron microscopy (SEM) operating at an accelerating voltage of 15 kV. Prior to imaging, the samples underwent a gold coating process using plasma sputter coating.

#### 2.4.2 Fourier-transform infrared spectroscopy (FTIR)

FTIR was used to analyze the chemical composition and interaction between chemical groups present in raw propolis, CS, PEO, as well as treated-CS/PEO and propolis-CS/PEO nanofiber scaffolds. The spectral analysis was conducted over the range of 4000–500 cm^−1^.

#### 2.4.3 Contact angle test

The surface wettability of the electrospun nanofiber scaffolds was assessed by a contact angle measuring drop shape analyzer. The reported values represent the average contact angle obtained from three samples tested for each analysis.

#### 2.4.4 Water absorption capacity

To evaluate the water absorption capacity of CS/PEO nanofiber scaffolds with and without propolis, the following procedure was followed:

1. Cut pieces of nanofibers were weighed to obtain their dry weight (Wd).
2. The nanofiber pieces were then immersed in distilled water at 37 °C.
3. After soaking for 4, 10, and 24 h, the excess water of swollen nanofibers were removed by filter paper and weighed (Ws).
4. The following equation was used to measure the water absorption percentage of nanofibers:

*water absorption percentage*$=\frac{Ws-Wd}{Wd}$ ˣ 100 Eq 1

#### 2.4.5 MTT assay

The viability of human foreskin fibroblast cell line (HFFF2) NCBI Code: C163 was obtained from National Cell Bank of Iran, Pasteur Institute of Iran was evaluated at 24 and 72 h using the MTT assay. Samples were prepared by cutting them into 1 × 1 cm pieces and sterilizing them with Ultraviolet *(*UV*)* radiation for 1 h and washing with PBS (Gibco, USA). The samples transferred to individual 96-well tissue culture plates. Cells (1 × 10^5^) were then seeded onto them and incubated at 37 °C in a humidified 5 % CO2 incubator. Next, 60 μL of 0.5 mg/ml MTT solution (Gibco, USA) was added to each well and incubated for 3 h at 37 °C. After this, 100 μL of DMSO (Sigma-Aldrich, USA) was added to dissolve the formazan crystals, followed by gentle shaking for about 10 min. The optical density was measured at 575 nm using a 96-well plate reader.

#### 2.4.6 Antibacterial test

To perform the antibacterial test, strains of *S. aureus* (ATCC 25923), *S. epidermidis* (ATCC 12228), *E. faecalis* (ATCC 29212), and *P. aeruginosa* (ATCC 27853) were cultured.

##### 2.4.6.1 Minimum inhibitory concentration (MIC) determination

The antibacterial efficacy of propolis extract was tested using the standard broth dilution method. Serial ten-fold dilutions were made from an initial concentration of 125 mg propolis in 2.5 ml Mueller Hinton broth (Merck, Germany) to determine the MIC. A control group with inoculated broth without propolis was incubated for 24 h at 37 °C. The MIC was identified as the lowest concentration with no visible growth, confirmed by visual assessment of turbidity before and after incubation.

##### *2.4.6.2* Minimum bactericidal concentration *(MBC) determination*

After establishing the MIC, samples from the wells without visible bacterial growth were cultured on Mueller Hinton agar (Merck, Germany) and incubated at 37 °C for 24 h. The MBC was determined as the lowest concentration that killed 99.9 % of the bacteria, based on the absence of bacterial colonies after incubation.

#### 2.4.7 Determination of anti-biofilm activity

##### 2.4.7.1 Biofilm formation assay by tissue culture plate

25 cc of TSB (Merck, Germany) containing 2 % glucose was poured into a sterile 96-well tissue culture plates using a sterile pipette, and then weighed propolis (maximum propolis concentration of 5 mg/ml) was added to it, then shaken for several hours to dissolve the propolis into the culture medium. Then the TSB culture medium containing 2 % glucose was diluted according to the MIC determined for each bacteria.

156.25 mg/ml of propolis was dissolved in 25 cc of TSB containing 2 % glucose. Then it was diluted again according to the minimum inhibitory concentration of each bacteria. 100 μl of the culture medium containing propolis was poured into the wells and then 100 μl of the turbidity suspension equivalent to 0.5 McFarland was added to the wells. This was repeated 3 times for each bacteria.

Then it was incubated for 24 h, after emptying the contents of the plate, it was washed twice with phosphate buffer and after drying, 95 % methanol was added to each well for 15 min to stabilize and then they were stained with 1 % Crystal Violet (Merck, Germany) for 5 min. Then it was washed with sterile distilled water and then 100 μL of 33 % acetic acid was added to each well and then Optical density (OD) was read with an ELISA reader (STAT FAX, USA) at a wavelength of 570 nm.

A well containing a bacterial suspension equivalent to 0.5 McFarland turbidity serves as a positive control, and a well containing TSB culture medium diluted according to the MIC determined for each bacteria serves as a negative control.

The results were reported in four forms based on the power of biofilm formation) inability to form biofilm, weak, medium and strong (.OD ≤ ODc: inability to form biofilm, ODc < OD ≤ 2 ODc: weak, 2 ODc < OD ≤ 4 ODc: medium, 4 ODc < OD: strong)

##### 2.4.7.2 Congo red agar method

To perform this test, Brain Heart Infusion Agar (Merck, Germany) containing 50 g/L sucrose, and 0.8 g/L Congo red stain (Merck, Germany) was used. Then, according to the MIC, the 5 mg/ml of propolis was weighed for each bacteria and added to the solution and placed in a water bath at 60 °C until the propolis dissolved in it. And after dissolving, it was added to three plates (separately for each bacteria). Then, the bacteria were cultured on them in a linear manner and placed in an incubator for 24 and 48 h. It was repeated 3 times for each bacteria.This was repeated 3 times for each of the bacteria. The formation of a black, rough colony on the culture medium indicates that the bacteria have formed a strong biofilm. The formation of a red, rough colony on the culture medium indicates that the bacteria have formed a medium-strong biofilm. The formation of a smooth, red colony on the culture medium indicates that the bacteria have formed a weak biofilm (no biofilm).

### 2.5 Evaluation of antibacterial effect by disk diffusion test

The disk diffusion method is a standard laboratory technique utilized to evaluate the antimicrobial properties of various substances, including antibiotics, disinfectants, and natural extracts. In this experiment, strains of *S. aureus*, *S. epidermidis*, *E. faecalis*, and *P. aeruginosa* bacteria were employed. First, nanofiber discs with a diameter of 6 mm were cut using a biopsy punch, then the discs were separated from the foil. The discs were sterilized by UV radiation. Mueller Hinton Agar medium was used for the disk diffusion test. A suspension of the desired bacteria was prepared to 0.5 McFarland turbidity and then it was cultured on Mueller Hinton Agar using a sterile swab in a wide-spread manner. After placing the discs in an incubator at 35 °C for 18 h (78). After this period, the inhibition zone diameter around the disc was measured with a ruler. This was repeated 3 times for each bacteria. This was repeated 3 times for each bacteria. In this test, chitosan discs alone, chitosan discs containing different concentrations of propolis (1 %, 2.5 %, 5 %, 10 %, and 20 %), and antibiotic discs specific to each bacterium selected based on the Clinical & Laboratory Standards Institute (CLSI) table were used as positive controls.

### 2.6 Statistical analysis

The results were evaluated by SPSS 2022 (SPSS Inc., Chicago, IL, USA). results was presented in descriptive form (including frequency, percentage, size, index deviation). The diameter of nanofibers was measured using image j software.

## 3 Results and discussion

### 3.1 SEM image analysis

**Fig. 1:**
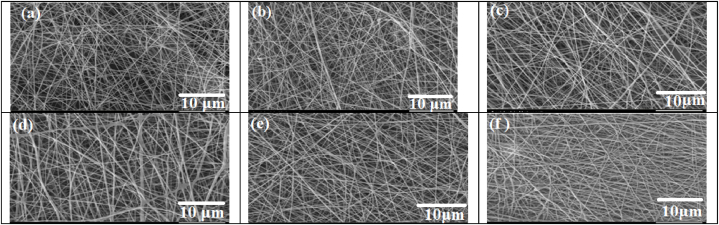
Morphologies of the electrospun nanofibers: Chitosan/PEO(a); Chitosan/PEO/Propolis 1 % (b); Chitosan/PEO/Propolis 2.5 % (c); Chitosan/PEO/Propolis5% (d); Chitosan/PEO/Propolis10 % (e); and Chitosan/PEO/Propolis 20 % (f).

The morphology of chitosan/PEO and chitosan/PEO/propolis nanofibers at concentrations of 1, 2.5, 5, 10, and 20 wt% was analyzed using SEM. The SEM images revealed that all samples exhibited beadless, tubular nanofibers. The average diameter of the chitosan/PEO nanofibers was measured to be 168.074 nm. Previous research has reported diameter ranges for chitosan/PEO nanofibers between 60 and 200 nm. For example, Abid et al. found an average diameter of about 116 nm for chitosan/PEO nanofibers [25]. Dorraki et al. reported fiber's diameter ranges of Chitosan/PEO from 110 to 240 nm [26]. Pakravan et al. reported the average diameter of chitosan/PEO nanofiber was 170 ± 21 nm for 4 wt % PEO concentration [27]. Surendhiran et al. observed chitosan/PEO nanofibers (50:50) with average diameters of 253 ± 23 nm [28]. It has been noted in previous studies that incorporating natural antimicrobial agents into electrospun fibers tends to increase their diameter [29]. In our investigation, we observed that the average diameters of chitosan/PEO/Propolis (at concentrations of 1 %, 2.5 %, 5 %, 10 %, and 20 %) increased to 188.605 nm, 198.294 nm, 203.843 nm, 210.052 nm, and 266.910 nm, respectively. Despite this increase, the chitosan/PEO/propolis nanofibers maintained a uniform surface, devoid of beads, and retained their tubular structure. This finding aligns with the results of Ceylan et al., who also reported a uniform surface morphology in PVA/chitosan/propolis (0.25 v/v) nanofibers [30]. Additionally, Moradkhannejhad et al. found that the average diameter of zein/propolis nanofibers increased from 264 to 419 nm when incorporating ethanol extract propolis ranging from 0 to 40 wt%. Additionally, all formulations displayed ribbon-like fibers with a relatively smooth surface, devoid of drug crystals [31] (see Fig. 1).

### 3.2 FT-IR spectroscopy of nanofibers

**Fig. 2:**
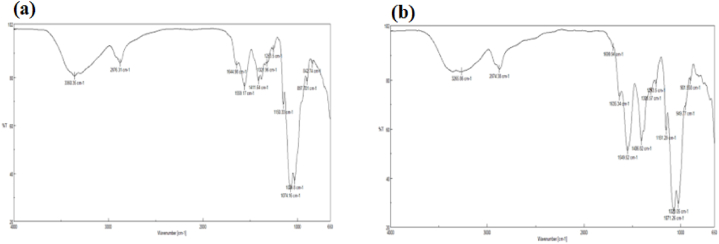
FT-IR spectrum of: Fig. 2a) Chitosan/PEO nanofibers Fig. 2b) Chitosan/PEO/Propolis nanofibers.

FT-IR spectra were utilized to identify the functional groups and intermolecular interactions within the physical mixture. The spectra for both chitosan/PEO nanofibers and propolis-loaded chitosan/PEO nanofibers are presented in Fig. 2. In Fig. 2a, the band at 3360.36 cm⁻^1^ corresponds to the stretching vibrations of the O-H and N-H bonds in chitosan, as well as the O-H bond in PEO [32]. As shown in Fig. 2b, the broad peak around 3266.86 cm⁻^1^ corresponds to the stretching vibrations of the O-H and N-H bonds in chitosan, the hydrogen-bonded O-H stretching of phenolic hydroxyl groups in propolis, and the O-H bond in the PEO backbone. Additionally, the bands observed at 2874.36 cm⁻^1^ are attributed to the asymmetric stretching of the CH2 groups in propolis [33]. The peak at 1265 cm⁻^1^ is associated with the C-O stretching vibrations, which can be attributed to the hydroxyflavonoids present in the propolis extract [34,35]. The peaks observed between 1151.29 cm⁻^1^ and 901.558 cm⁻^1^ can be attributed to the stretching vibrations of the C-C groups. The FTIR results suggest physical interactions between propolis, chitosan, and PEO in the final sample, indicating the successful incorporation of propolis in the final product.

### 3.3 Water contact angle test

**Fig. 3:**
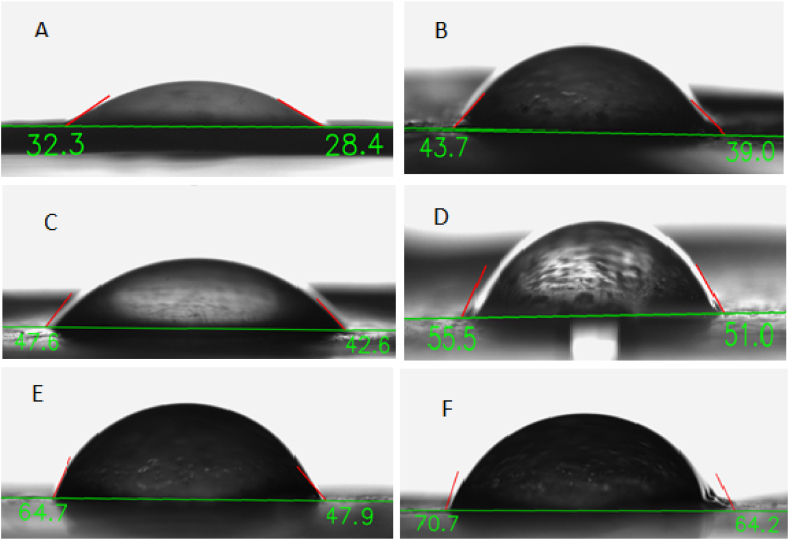
Water contact angle of chitosan/PEO (A), Chitosan/PEO/Propolis 1 % (B); Chitosan/PEO/Propolis 2.5 % (C); Chitosan/PEO/Propolis5% (D); Chitosan/PEO/Propolis10 % (E); and Chitosan/PEO/Propolis 20 % (F). (As the percentage of propolis increases, the hydrophilicity decreases, but in the chitosan-propolis 20 % scaffold, the contact angle is 70°, and this hydrophilicity may be suitable for cell attachment, growth, and adhesion.)

Typically, if the water contact angle measures less than 90°, the solid surface is considered hydrophilic. While, an angle exceeding 90° suggests a hydrophobic surface [36]. The Chitosan/PEO nanofibers exhibited a water contact angle of approximately 32.3°, which is below 90°, indicating a hydrophilic surface. Based on Fig. 3, the addition of varying amounts of propolis resulted in a slight increase in the water contact angle, ranging from 43 to 70°. However, this increase was not significant enough to alter the hydrophilicity of the nanofibers. Consequently, the electrospun nanofibers retained their hydrophilic nature even after the incorporation of propolis. Our findings align with those of Ulag et al., who reported that the addition of propolis decreased the hydrophilicity of the PVA/GEL composite, confirming the hydrophobic nature of propolis [37]. In another study conducted by Bilginer et al., contact angles ranging from 49.58 ± 7.8 to 74.46 ± 6.87 were observed for PVA scaffolds integrated with propolis, thereby confirming the hydrophobic nature of propolis (38). The water contact angle property of less than 90° indicates the favorable hydrophilicity of the nanofibers. Consequently, Chitosan/PEO nanofibers loaded with propolis would be suitable for absorbing wound exudates and retaining moisture, thus offering a novel type of wound dressing.

### 3.4 Swelling properties

**Fig. 4:**
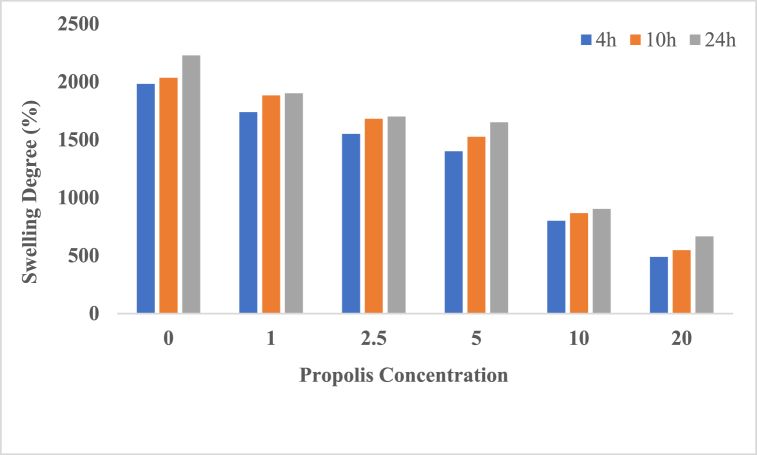
Swelling degree (%) of Chitosan/PEO nanofiber scaffolds with different amounts Propolis (0, 1, 2.5, 5, 10 and 20 %) (As the percentage of propolis increases, their ability to absorb water in scaffolds increases, and this is considered an advantage in these scaffolds.).

The absorption of wound secretions plays a crucial role in accelerating wound treatment. Therefore, fluid uptake capability is a key parameter in the design of wound dressings. The swelling behavior of Chitosan/PEO, both with and without Propolis, in PBS at 25 °C was assessed. According to Fig. 4, chitosan/PEO nanofibers exhibited significant swelling abilities, expanding by more than 2000 % (w/w) after exposure to PBS for 4, 12, and 24 h. This significant swelling can be attributed to the presence of PEO, which acts as an osmotic agent [38]. In a comparable study, Stie et al. reported that chitosan/PEO nanofibers exhibited a swelling ratio of 1132 ± 36 %, thus confirming the substantial swelling behavior characteristic of chitosan/PEO nanofibers [39]. The incorporation of propolis into Chitosan/PEO nanofibers results in a reduction in the swelling ratio. Specifically, a concentration of 20 % propolis led to a significant decrease in the swelling of chitosan/PEO nanofibers. Consistent with various studies, increasing propolis concentration tends to decrease the swelling ratio. For instance, Ceylan et al. demonstrated a similar trend, where higher concentrations of propolis resulted in lower swelling ratios ([30]. In another study by Suriyatem et al., it was found that the swelling rate of rice starch/carboxymethyl chitosan films decreased gradually from 3764 % to 211 % as the concentration of propolis increased from 0 % to 10 %. This decrease in swelling capacity could be attributed to the increase in hydrophobic groups, such as aromatic rings and polyphenols found in propolis [40]. Khodabakhshi et al. demonstrated that an increase in propolis concentration (from 10 % to 30 %) in polyurethane/propolis foams resulted in decreased water absorption, ranging from 243 ± 15 % to 207 ± 14 % [41]. The findings by Du et al. suggested that with increasing propolis concentration, the hydrophilicity and water uptake content of propolis-enriched silk fibroin-gelatin composite nanofibers decreased. This trend could be attributed to the presence of hydrophobic groups, such as fatty acids and terpenes, present in propolis [42]. The water retention capacity of a trauma dressing plays a crucial role in maintaining a moist local environment, which is essential for preventing wound dryness. Furthermore, it helps prevent wound infection by effectively absorbing fluid and exudate from the wound bed [[43], [44], [45]]. Indeed, despite the inherent hydrophobic nature of propolis, propolis-loaded chitosan/PEO nanofibers exhibit a notable water absorption capacity. This characteristic makes them highly promising for use as wound dressings, as they effectively absorb secretions and maintain a moist environment, which is crucial for promoting wound healing.

### 3.5 Cell viability

**Fig. 5:**
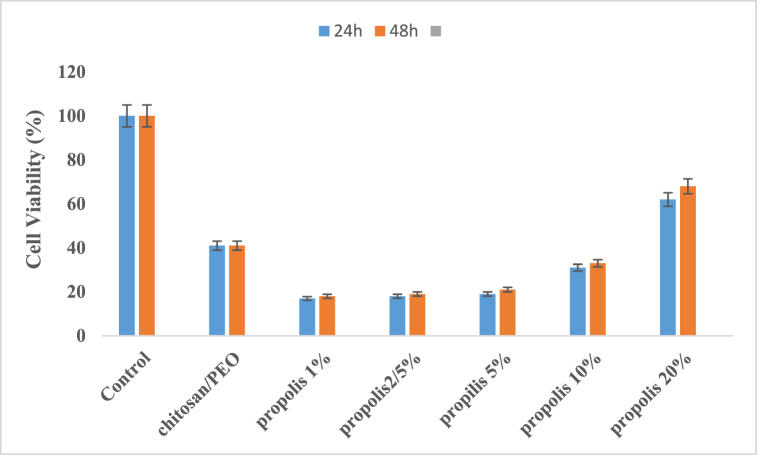
Cell viabilitty of Chitosan/PEO loaded by different concentration of propolis (1 %, 2.5 %, 5 %, 10 %, and 20 %) (After 24 h, the survival coefficient of the chitosan scaffold was 13 %, and the chitosan-propolis scaffolds with concentrations of 1 %, 2.5 %, 5 %, 10 %, and 20 % were 17 %, 18 %, 19 %, 31 %, and 62 %, respectively. This test showed that cell survival increased with increasing propolis. After 48 h, the survival coefficient of the chitosan scaffold was 15 %, and the chitosan-propolis scaffolds with concentrations of 1 %, 2.5 %, 5 %, 10 %, and 20 % were 18 %, 19 %, 21 %, 33 %, and 68 %, respectively. This test showed that cell survival increased with increasing propolis.

MTT test was performed to investigate the biocompatibility and viability of different concentrations of nanofibers on human fibroblast cells (HFFF2) after 48 and 72 h in vitro. Fig. 5 demonstrates a direct correlation between escalating propolis concentration and heightened cell viability.

After 48 h, the results of the cell viabilitty exposed to chitosan scaffolds were 13 % and the results of the 1 %, 2.5 %, 5 %, 10 % and 20 % concentrations of chitosan-propolis scaffolds were 17 %, 18 %, 19 %, 31 % and 62 %, respectively. The result of cell viabilitty exposed to the positive control (including human fibroblast cells without propolis) was 40 %. The results of this test showed that the percentage of cell viabilitty increased with increasing propolis concentration.

After 72 h, the results of the percentage of cell viabilitty exposed to 15 % chitosan scaffold and concentrations of 1 %, 2.5 %, 5 %, 10 % and 20 % chitosan-propolis scaffolds were reported as 18 %, 19 %, 21 %, 33 % and 68 %, respectively. The result of the cell viabilitty exposed to the positive control (including human fibroblast cells without propolis) was reported as 41 %. The results of this test showed that the percentage of cell viabilitty increased with increasing propolis concentration.

Similarly, in a study by Eloureiro et al., chitosan/collagen membranes containing with red propolis extract exhibited cell viability below the 70 % threshold [46]. In other study Oliveira et al., showed the cell viability was lower than 70 % for PVA–propolis samples [35]. The results of the MTT test showed that the percentage of cell viability increased with increasing propolis concentration. Based on these results, it can be concluded that propolis is not only biocompatible but also stimulates cell growth and division.

### 3.6 Antibacterial effect

Table 1 presents the MIC and MBC of raw propolis against strains of *S. aureus*, *S. epidermidis*, *E. faecalis*, and *P. aeruginosa*. The findings indicate that propolis exhibits antibacterial effects, with MIC values of 0.039 mg/mL against *S. aureus*, 0.156 mg/mL against *S. epidermidis*, 0.078 mg/mL against *E. faecalis*, and 2.5 mg/mL against *P. aeruginosa*. Several studies have indicated that a significant portion of chemical constituents found in propolis, such as flavonoids and cinnamic acid derivatives, possess antibacterial properties [[47], [48], [49], [50]]. As an illustration, Ota et al. attributed the antibacterial effects of propolis to its polar compounds, particularly phenols, including flavonoids, phenolic acids, and their esters [51]. Consistent with earlier research, this study's findings revealed that Gram-positive bacteria exhibited greater sensitivity to propolis compared to Gram-negative bacteria [52,53]. According to Abdulkhani et al., propolis demonstrated antibacterial activity against all Gram-positive strains examined, especially with notable susceptibility on *B. cereus*, B. anthracis and *S. aureus* [54]. In the research conducted by Khoshnevisan et al., propolis exhibited significant antibacterial activity against *S. aureus* and S. epidermidis, while demonstrating a comparatively weaker effect against Gram-negative bacteria [34]. This less sensitivity of Gram-negative bacteria to propolis may result from variations in the chemical structure of their cell walls, hindering propolis penetration into the bacterial cells [55]. The MIC and MBC results confirm propolis' significant antibacterial efficacy.

**Table 1:** MIC and MBC of propolis against gram positive and gram negative bacteria **(The minimum inhibitory concentration (MIC) of propolis against *S. aureus* was 0.039 mg/ml, *S. epidermidis* was 0.156 mg/ml, *E. faecalis* was 0.078 mg/ml, and *P. aeruginosa* was 2.5 mg/ml. The Minimum bactericidal concentration (MBC) of propolis against *S. aureus* was 0.078 mg/ml, *S. epidermidis* was 0.312 mg/ml, *E. faecalis* was 0.156 mg/ml, and P. aeruginosa was 5 mg/ml)**.

Strains	MIC (mg/ml)	MBC (mg/ml)
*S. aureus*	0.039	0.078
*S. epidermidis*	0.165	0.312
*E. faecalis*	0.078	0.156
*P. aeruginosa*	2.5	5

### 3.7 Anti-biofilm activity of propolis and CS/PEO -propolis by Congo red agar plate method

**Fig. 6:**
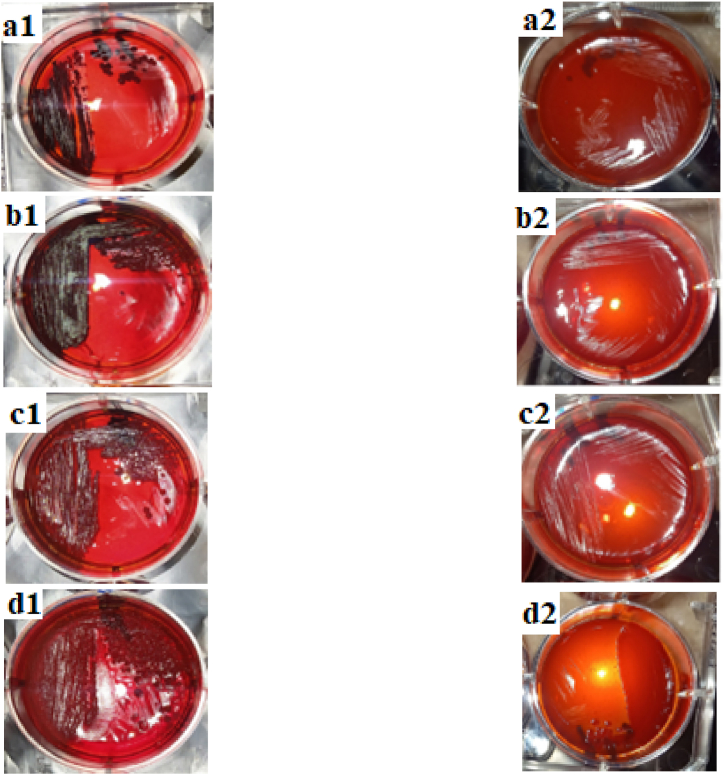
Evaluation of the antibiofilm activity of propolis by Congo red agar plate method. The appearance of black colonies (a1,b1,c1 and d1) indicates the exopolysaccharide production by *S. aureus*, *S. epidermidis*, *E. faecalis* and *P. aeruginosa* bacteria, respectively. Whereas the addition of propolis block the exopolysaccharide secretion by bacteria and weakened their growth (a2,b2,c2 and d2). *S. aureus* grew on Congo red medium without propolis and formed a strong biofilm (positive control), while it did not grow on Congo red medium containing 0.039 mg/ml of propolis. *S. epidermidis* grew on Congo red medium without propolis and formed a strong biofilm (positive control), while it did not grow on Congo red medium containing 0.156 mg/ml of propolis. *E. faecalis* grew on Congo red medium without propolis and formed a strong biofilm (positive control), while it did not grow on Congo red medium containing 0.078 mg/ml of propolis. *P. aeruginosa* grew on Congo red medium lacking propolis and formed a moderate biofilm (positive control), while on Congo red medium containing 2.5 mg/ml of propolis. It grew and formed a weak biofilm.

To assess the anti-biofilm potential of propolis, we utilized the Congo red agar plate method against strains of *S. aureus*, *S. epidermidis*, *E. faecalis*, and *P. aeruginosa*. The presence of dry crystalline black colonies on the plate indicates the secretion of exopolysaccharides, which facilitate microbial adhesion and biofilm formation, and protects them from unfavourable environmental factors. Based on the findings illustrated in Fig. 6, plates lacking propolis supplementation exhibited the emergence of dry crystalline black colonies in cultures of *S. aureus*, *S. epidermidis*, *E. faecalis*, and *P. aeruginosa*. Conversely, when propolis was present in the plates, the growth of *S. aureus*, *S. epidermidis*, and *E. faecalis* strains occurred, yet the absence of dry crystalline black colonies indicated propolis's inhibitory effect on biofilm formation. However, on the plate containing *P. aeruginosa* supplemented with propolis, the organism did grow, but along with a moderate inhibition of dry crystalline black colonies. These findings suggest that the anti-biofilm effect of propolis was particularly sensitive against Gram-positive bacteria.

Anti-biofilm characteristics of the CS/PEO loaded by 0.039, 0.078, 0.156, 0.312, 0.625,1.25, 2.5 and 5 mg propolis/mL measured against *Enterococcus faecalis*, *Pseudomonas aeruginosa*, *Staphylococcus aureus* and *Staphylococcus epidermidis*.

*S. aureus* grew on Congo red medium without propolis and formed a strong biofilm (positive control), while it did not grow on Congo red medium containing 0.156 mg/mL propolis loaded onto CS/PEO nanofiber.

*S. epidermidis* grew on Congo red medium without propolis and formed a strong biofilm (positive control), while it did not grow on Congo red medium containing 0.625 mg/mL propolis loaded on to CS/PEO nanofiber.

*E. faecalis* grew on Congo red medium without propolis and formed a strong biofilm (positive control), while it did not grow on Congo red medium containing 0.312 mg/mL propolis loaded on to CS/PEO nanofiber.

*P. aeruginosa* grew on Congo red medium lacking propolis and formed a moderate biofilm (positive control), while it did not grow on Congo red medium containing 5 mg/mL propolis loaded on to CS/PEO nanofiber.

### 3.8 Anti-biofilm activity of propolis and CS/PEO -propolis by tissue culture plate method

Table 2 presents the results of the anti-biofilm activity of propolis using the Tissue Culture Plate method. The impact of propolis on biofilm formation varied among the tested Gram-positive and Gram-negative strains. The susceptibility to propolis was notably high in the examined Gram-positive bacteria (*S. aureus*, *S. epidermidis*, and *E.faecalis*), indicating potent anti-biofilm activity. Conversely, for the tested Gram-negative bacterium (*P. aeruginosa*), the sensitivity to propolis was comparatively lower, resulting in moderate anti-biofilm activity. For *S. aureus*, the concentration of propolis used in the Tissue Culture Plate method was 0.039 mg/ml, and the biofilm formed was weak. For *S. epidermidis*, the concentration of propolis used in the Tissue Culture Plate method was 0.156 mg/ml, and the biofilm formed was weak. For *E. faecalis*, the concentration of propolis used in the Tissue Culture Plate method was 0.078 mg/ml, and the biofilm formed was weak. For *P. aeruginosa*, the concentration of propolis used in the Tissue Culture Plate method was 2.5 mg/ml, and the biofilm formed was medium.

**Table 2:** Anti-biofilm activity of propolis by Tissue Culture Plate method.

Bacteria	Values		BIC_50_
	A (nm)	P (%)	
*S. aureus*	0.463	47.73	0.039
*S. epidermidis*	0.313	20.127	0.156
*E. faecalis*	0.534	38.76	0.078
*P. aeruginosa*	0.832	51.156	2.5

A - Absorbance of biofilm.

P - Percentage of biofilm growth, Biofilm inhibitory concentration (BIC_50_) in mg/ml.

For *S. aureus*, the concentration of CS/PEO -propolis used in the Tissue Culture Plate method was 0.156 mg/ml, and biofilm did not form. For *S. epidermidis*, the concentration of CS/PEO -propolis used in the Tissue Culture Plate method was 0.625 mg/ml, and the biofilm formed was weak. For *E. faecalis*, the concentration of CS/PEO -propolis used in the Tissue Culture Plate method was 0.312 mg/ml, and the biofilm formed was weak. For *P. aeruginosa*, the concentration of CS/PEO -propolis used in the Tissue Culture Plate method was 5 mg/ml, and the biofilm formed was medium (Table 3).

**Table 3:** Anti-biofilm activity of CS/PEO -propolis by Tissue Culture Plate method.

Bacteria	Values		BIC_50_
	A (nm)	P (%)	
*S. aureus*	0.513	49.256	0.156
*S. epidermidis*	0.424	27.84	0.625
*E. faecalis*	0.651	34.52	0.312
*P. aeruginosa*	0.984	60.68	5

A - Absorbance of biofilm.

P - Percentage of biofilm growth, Biofilm inhibitory concentration (BIC_50_) in mg/ml.

### 3.9 Antibacterial activity by the disk diffusion method

**Fig. 7:**
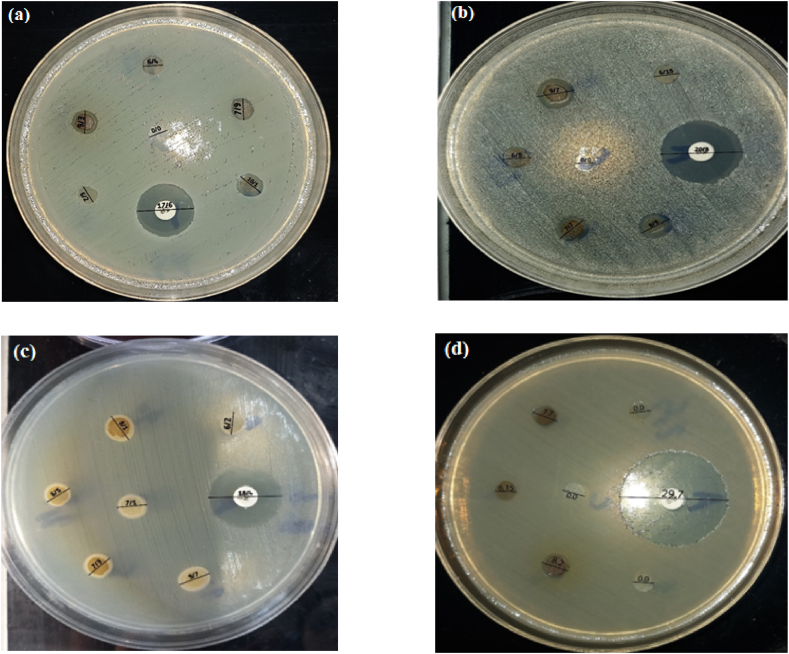
The antibacterial activity of chitosan/PEO coated with 1–20 % v/v of propolis against *S. aureus* (a), *S. epidermidis* (b), *E.faecalis* (c) and *P. aeruginosa* (d).

The antibacterial activity of chitosan/PEO coated with varying concentrations (1–20 % v/v) of propolis against *S. aureus*, *S. epidermidis*, *E. faecalis*, and *P. aeruginosa* was examined using the disk diffusion method. Table 4 presents the results, indicating that as the concentration of propolis increased, its inhibitory effectiveness also increased. Based on the inhibitation zone illustrated in Fig. 7, the antibacterial effectiveness of Chitosan/PEO incorporating propolis was noticeable against strains of *S. aureus*, *S. epidermidis*, and *E. faecalis*, which are Gram-positive bacteria. Notably, *S. aureus* displayed the highest susceptibility to propolis among these tested bacterial strains. In this investigation, propolis-loaded Chitosan/PEO exhibited a moderate impact on the *P. aeruginosa* strain, a Gram-negative bacterium. Notably, at lower concentrations of propolis (1 % and 2.5 %), the Chitosan/PEO nanofiber did not produce discernible bacterial inhibition zones.

**Table 4:** The Antibacterial Activity of Chitosan/PEO nanofiber with different concentration of propolis using disc diffusion method.

Concentration of propolis	inhibition zone diameter (mm)				
	1 %	2.5 %	5 %	10 %	20 %
*S. aureus*	6.2	6.4	7.9	9.3	10.1
*S.epidermidis*	6.15	6.5	7.7	8.9	9.7
*E. faecalis*	6.5	7.1	7.3	8.1	9.7
*P. aeruginosa*	0.0	0.0	6.15	7.7	8.2

Inhibition zone diameter of 6.2, 6.4, 7.9, 9.3, and 10.1 mm were formed around chitosan-propolis scaffold disks at concentrations of 1 %, 2.5 %, 5 %, 10 %, and 20 % against *S. aureus*, respectively. (Vancomycin antibiotic disk as positive control)

Inhibition zone diameter of 6.15, 6.5, 7.7, 8.9, and 9.7 mm were formed around the chitosan-propolis scaffold disks with concentrations of 1 %, 2.5 %, 5 %, 10 %, and 20 % against *S. epidermidis*, respectively. (Vancomycin antibiotic disk as positive control)

**Inhibition zone diameter of 6.5, 1.7, 3.7, 1.8 and 9.7 mm were formed around the chitosan-propolis scaffold disks with concentrations of 1 %, 2.5 %, 5 %, 10 % and 20 % against** *E. faecalis* **, respectively. (Gentamicin antibiotic disc as positive control)**

Inhibition zone diameter of 6.5, 1.7, 3.7, 1.8 and 9.7 mm were formed around the chitosan-propolis scaffold disks with concentrations of 1 %, 2.5 %, 5 %, 10 % and 20 % against *P. aeruginosa*, respectively. (Imipenem antibiotic disk as positive control)

## 4 Conclusion

Our goal in this study is to build an optimal scaffold in terms of cellular, physical and mechanical response to be used as wound dressing and antibacterial cases. In this study, chitosan-propolis scaffolds with different concentrations were made and antibacterial, cellular and biocompatibility evaluations were also performed for it. In this study, propolis was extracted with 70 % ethanol so that the research done in Brazil by Veiga et al. The concentration of artypillin C is higher in propolis ethanolic extract compared to hexane extract. These extracts also showed high antibacterial activity on MRSA (methicillin-resistant *Staphylococcus aureus*) [56]. This scaffold was well prepared and has a good shape and morphology, so that the results of the electron microscope showed that the nanofibers have a good diameter, so that the diameter of the chitosan nanofibers is 168 nm found In 2013, Gu BK et al. conducted a study entitled "Manufacturing ultrasonic chitosan nanofiber mat with enlarged porosity for use as hemostatic material" which showed that the diameter of nanofibers increased by adding another polymer to chitosan [57].

Also, in a study conducted by Chen et al., in 2010, a study titled "Electrospun collagen-chitosan nanofibers: a biomimetic extracellular matrix for endothelial cells and smooth muscle cells" showed that adding collagen to chitosan increased the diameter of nanofibers [58]. FTIR test also confirmed that propolis is present inside the scaffold and has good linkage and peaks. In the degradability test, it showed that the chitosan scaffold has good stability, and with the addition and increase of propolis, the stability of the scaffolds increases. In 2016, Mengistu Lemma and colleagues conducted a study titled "Preparation of pure and stable chitosan nanofibers by electrospinning in the presence of polyethylene oxide", which showed that chitosan nanofibers have good stability [59].

In the drug release test, it showed that between 12 and 24 h the maximum release of propolis from the scaffold. In the contact angle test, it showed that with the increase of propolis in the scaffolds, the amount of hydrophilicity of the scaffolds decreased. Abdukhani et al. conducted a study in 2017 entitled "Evaluation of antibacterial activity of cellulose/polylactic acid nanofiber composites coated with propolis ethanolic extract" showing that increasing propolis in nanofibers increases their hydrophobicity, which It can be caused by phenolic compounds, fatty acids and terpenes [54]. Also, the biodegradability test showed that as the amount of propolis increases, the stability of the scaffolds increases, which can be caused by the hydrophobic property of propolis.

In this study, the effect of propolis on the biofilm of *S. aureus*, *S. epidermidis*, *E. faecalis* and *P. aeruginosa* was investigated and it was shown that propolis has the greatest effect on the biofilm of *S. aureus*, *S. epidermidis* and *E. faecalis*, so as to prevent the growth of bacteria. But against P*. aeruginosa*, the effect of propolis is low, so that it reduced the strength of the biofilm. In 2021, Calderón et al. conducted a study titled "Antifungal and anti-biofilm activity of Spanish propolis extract." This study showed that propolis is effective on the biofilm of various bacteria, which is in line with the observations of this study [59]. In 2011, Kashi and his colleagues conducted a study titled "Investigation of the antibacterial effect of iranian propolis on oral microorganisms", which concluded that Iranian propolis is a valuable source for controlling oral biofilm and subsequently causing tooth decay.

In this study, the minimum inhibitory concentration (MIC) of *S. aureus*, *S. epidermidis*, *E. faecalis* and *P. aeruginosa* were 0.016 mg/ml, 0.032 mg/ml, 0.032 mg/ml, and 512/512, respectively. 0 mg/ml was reported [60]. The results of the present study also showed that the MIC for the bacteria in question is 0.039 mg/ml, 0.156 mg/ml, 0.078 mg/ml, and 2.5 mg/ml, respectively. It is completely in line with the results of Uzel that the results of both studies show the sensitivity of gram-positive bacteria to gram-negative bacteria, which is due to the difference in the structure of the membrane and cell wall of bacteria.

1801/5000.

In this study, the minimum inhibitory concentration (MIC) of *S. aureus*, *S. epidermidis*, *E. faecalis* and *P. aeruginosa* were 0.008 mg/ml, 0.020 mg/ml, 0.016 mg/ml, 120 0.0 mg/ml was reported [61]. The results of the present study also showed that the MIC for the bacteria in question is 0.039 mg/ml, 0.156 mg/ml, 0.078 mg/ml, and 2.5 mg/ml respectively, which are completely It is the same with Przybyłek's results that the results of both studies show the sensitivity of gram-positive bacteria to gram-negative bacteria, which is due to the difference in the membrane and cell wall structure of bacteria [10].

In 2020, Eskandarinia et al. conducted a study titled "Properties of a new double-layer wound dressing consisting of a dense polyurethane/propolis membrane and biodegradable polycaprolactone/gelatin nanofiber scaffold" epidermidis and *P. aeruginosa* showed that PU/EEP membrane has the best antibacterial activity against *S. aureus*, which is about 5.10, while *S. epidermidis* 8.1 and *P. aeruginosa* did not form a halo of non-growth [62]. The results of the present study also show *S. aureus* formed the largest inhibition zone diameter of non-growth among bacteria and *P. aeruginosa* also had the lowest inhibition zone diameter of non-growth. In the tests that investigated the effect of propolis on the biofilm of bacteria, it was shown that the effect of propolis on the biofilm of *S. aureus*, *S. epidermidis*, *E. faecalis*. It has a high but little effect against the gram-negative bacterium *P. aeruginosa*, which is consistent with the results of previous tests and studies conducted in this field. It also showed in the biocompatibility test that not only propolis does not cause cell death, but also acts as a stimulus for cell growth.

The primary aim of our research was to explore the efficacy of propolis extract, a traditional antibacterial agent, when incorporated into a Chitosan/PEO-based scaffold for the development of an innovative wound dressing. The morphology of Chitosan/PEO nanofiber revealed smooth and uniform nanofibers without visible beads, with a mean fiber diameter of 168.074 nm. Addition of propolis extract led to an increase in the average fiber diameter of Chitosan/PEO to 188.605–266.910 nm. FTIR analysis confirmed the presence of propolis extract in the Chitosan/PEO nanofibers. The hydrophilicity of the nanofiber scaffolds was verified through water contact angle measurements, which ranged from 43 to 70°. Furthermore, these nanofiber scaffolds exhibited substantial antibacterial activity against both gram-negative and gram-positive bacteria. In vitro studies indicated the non-toxic nature of the nanofiber scaffolds. This study highlights the potential of Chitosan/PEO nanofiber scaffolds loaded with propolis extract as effective antibacterial agents for promoting the wound healing process. The results of the MTT test showed that the percentage of cell viability increased with increasing propolis concentration. Based on these results, it can be concluded that propolis is not only biocompatible but also stimulates cell growth and division.

## CRediT authorship contribution statement

**Sayed Reza Ebrahimi:** Writing – original draft, Validation, Software, Resources, Methodology, Investigation, Conceptualization. **Mohammad Nikbakht:** Writing – original draft, Validation, Methodology, Investigation. **Milad Shahini Shams Abadi:** Writing – review & editing, Methodology, Formal analysis, Data curation. **Abolfazl Gholipour:** Writing – review & editing, Validation. **Mitra Bagheri:** Methodology, Investigation, Conceptualization. **Majid Validi:** Writing – review & editing, Supervision, Project administration, Methodology, Investigation.

## Declaration of competing interest

The authors declare that they have no known competing financial interests or personal relationships that could have appeared to influence the work reported in this paper.

## References

[1] Pfaller M. (1998). The SENTRY participants group. Bacterial pathogens isolated from patients with bloodstream infections: frequency of occurrence and antimicrobial susceptibility patterns from the SENTRY antimicrobial surveillance program (United States and Canada 1997). Antimicrob. Agents Chemother..

[2] Iwu M.W., Duncan A.R., Okunji C.O. (1999). New antimicrobials of plant origin. Perspectives on new crops and new uses ASHS Press, Alexandria, VA.

[3] Bush K. (2004). Antibacterial drug discovery in the 21st century. Clin. Microbiol. Infection.

[4] Rabbani M., Etemadifar Z., Karamifard F., Borhani M.S. (2016). Assessment of the antimicrobial activity of Melissa officinalis and Lawsonia inermis extracts against some bacterial pathogens. Comp. Clin. Pathol..

[5] Geyik F, Kaya S, Yılmaz DE, Demirci H, Akmayan I, Özbek T, Acar S (2024). Propolis-Loaded Poly (lactic-co-glycolic Acid) Nanofibers: An In Vitro Study. ACS omega.15;.

[6] Yaghoubi M., Gh G., Satari R. (2007). Antimicrobial activity of Iranian propolis and its chemical composition. Daru.

[7] Pascoal A., Feás X., Dias T., Dias L., Estevinho L., Kon K. (2014).

[8] Oryan A., Alemzadeh E., Moshiri A. (2018). Potential role of propolis in wound healing: biological properties and therapeutic activities. Biomed. Pharmacother..

[9] Czyżewska U., Konończuk J., Teul J., Drągowski P., Pawlak-Morka R., Surażyński A. (2015). Verification of chemical composition of commercially available propolis extracts by gas chromatography–mass spectrometry analysis. J. Med. Food.

[10] Przybyłek I., Karpiński T.M. (2019). Antibacterial properties of propolis. Molecules.

[11] Gucwa K., Kusznierewicz B., Milewski S., Van Dijck P., Szweda P. (2018). Antifungal activity and synergism with azoles of polish propolis. Pathogens.

[12] Ripari N., Sartori A.A., da Silva Honorio M., Conte F.L., Tasca K.I., Santiago K.B. (2021). Propolis antiviral and immunomodulatory activity: a review and perspectives for COVID-19 treatment. J. Pharm. Pharmacol..

[13] Asawahame C., Sutjarittangtham K., Eitssayeam S., Tragoolpua Y., Sirithunyalug B., Sirithunyalug J. (2015). Antibacterial activity and inhibition of adherence of Streptococcus mutans by propolis electrospun fibers. AAPS PharmSciTech.

[14] Rojczyk E., Klama-Baryła A., Łabuś W., Wilemska-Kucharzewska K., Kucharzewski M. (2020). Historical and modern research on propolis and its application in wound healing and other fields of medicine and contributions by Polish studies. J. Ethnopharmacol..

[15] Field C.K., Kerstein M.D. (1994). Overview of wound healing in a moist environment. Am. J. Surg..

[16] Zahedi P., Karami Z., Rezaeian I., Jafari S.H., Mahdaviani P., Abdolghaffari A.H. (2012). Preparation and performance evaluation of tetracycline hydrochloride loaded wound dressing mats based on electrospun nanofibrous poly (lactic acid)/poly (ϵ‐caprolactone) blends. J. Appl. Polym. Sci..

[17] Wang J., Vermerris W. (2016). Antimicrobial nanomaterials derived from natural products—a review. Materials.

[18] Andukuri A., Kushwaha M., Tambralli A., Anderson J.M., Dean D.R., Berry J.L. (2011). A hybrid biomimetic nanomatrix composed of electrospun polycaprolactone and bioactive peptide amphiphiles for cardiovascular implants. Acta Biomater..

[19] Rieger K.A., Birch N.P., Schiffman J.D. (2013). Designing electrospun nanofiber mats to promote wound healing–a review. J. Mater. Chem. B.

[20] de Queiroz Antonino R.S.C.M., Lia Fook B.R.P., de Oliveira Lima V.A., de Farias Rached R.Í., Lima E.P.N., da Silva Lima R.J. (2017). Preparation and characterization of chitosan obtained from shells of shrimp (Litopenaeus vannamei Boone). Mar. Drugs.

[21] Ayodele O., Okoronkwo A.E., Oluwasina O.O., Abe T.O. (2018). Utilization of blue crab shells for the synthesis of chitosan nanoparticles and their characterization. Songklanakarin J. Sci. Technol..

[22] Sandeep A., Sangameshwar K., Mukesh G., Chandrakant R., Avinash D. (2013). A brief overview on chitosan applications. Indo Am J Pharmacutical Res.

[23] Huang L., Nagapudi K., Apkarian R P., Chaikof E.L. (2001). Engineered collagen–PEO nanofibers and fabrics. J. Biomater. Sci. Polym. Ed..

[24] Singh Y.P., Dasgupta S., Nayar S., Bhaskar R. (2020). Optimization of electrospinning process & parameters for producing defect-free chitosan/polyethylene oxide nanofibers for bone tissue engineering. J. Biomater. Sci. Polym. Ed..

[25] Abid S., Hussain T., Nazir A., Zahir A., Ramakrishna S., Hameed M. (2019). Enhanced antibacterial activity of PEO-chitosan nanofibers with potential application in burn infection management. Int. J. Biol. Macromol..

[26] Dorraki N., Safa N.N., Jahanfar M., Ghomi H., Ranaei-Siadat S.-O. (2015). Surface modification of chitosan/PEO nanofibers by air dielectric barrier discharge plasma for acetylcholinesterase immobilization. Appl. Surf. Sci..

[27] Pakravan M., Heuzey M.-C., Ajji A. (2012). Core–shell structured PEO-chitosan nanofibers by coaxial electrospinning. Biomacromolecules.

[28] Surendhiran D., Li C., Cui H., Lin L. (2020). Fabrication of high stability active nanofibers encapsulated with pomegranate peel extract using chitosan/PEO for meat preservation. Food Packag. Shelf Life.

[29] Lin L., Xue L., Duraiarasan S., Haiying C. (2018). Preparation of ε-polylysine/chitosan nanofibers for food packaging against Salmonella on chicken. Food Packag. Shelf Life.

[30] Ceylan S. (2021). Propolis loaded and genipin-crosslinked PVA/chitosan membranes; characterization properties and cytocompatibility/genotoxicity response for wound dressing applications. Int. J. Biol. Macromol..

[31] Moradkhannejhad L., Abdouss M., Nikfarjam N., Mazinani S., Heydari V. (2018). Electrospinning of zein/propolis nanofibers; antimicrobial properties and morphology investigation. J. Mater. Sci. Mater. Med..

[32] Sadri M., Arab Sorkhi S. (2017). Preparation and characterization of CS/PEO/cefazolin nanofibers with in vitro and in vivo testing. Nanomedicine Research Journal.

[33] Do Nascimento T.G., Da Silva P.F., Azevedo L.F., Da Rocha L.G., de Moraes Porto I.C.C., Lima e Moura TFA. (2016). Polymeric nanoparticles of Brazilian red propolis extract: preparation, characterization, antioxidant and leishmanicidal activity. Nanoscale Res. Lett..

[34] Khoshnevisan K., Maleki H., Samadian H., Doostan M., Khorramizadeh M.R. (2019). Antibacterial and antioxidant assessment of cellulose acetate/polycaprolactone nanofibrous mats impregnated with propolis. Int. J. Biol. Macromol..

[35] Oliveira R.N., McGuinness G.B., Rouze R., Quilty B., Cahill P., Soares G.D. (2015). PVA hydrogels loaded with a Brazilian propolis for burn wound healing applications. J. Appl. Polym. Sci..

[36] Reisi-Vanani V., Hosseini S., Soleiman-Dehkordi E., Boroujeni S.N., Farzan M., Ebani V.V. (2023). Engineering of a core-shell polyvinyl alcohol/gelatin fibrous scaffold for dual delivery of Thymus daenensis essential oil and Glycyrrhiza glabra L. extract as an antibacterial and functional wound dressing. J. Drug Deliv. Sci. Technol..

[37] Ulag S., Ilhan E., Demirhan R., Sahin A., Yilmaz B.K., Aksu B. (2021). Propolis-based nanofiber patches to repair corneal microbial keratitis. Molecules.

[38] Patel V.R., Amiji M.M. (1996).

[39] Stie M.B., Gätke J.R., Wan F., Chronakis I.S., Jacobsen J., Nielsen H.M. (2020). Swelling of mucoadhesive electrospun chitosan/polyethylene oxide nanofibers facilitates adhesion to the sublingual mucosa. Carbohydr. Polym..

[40] Suriyatem R., Auras R.A., Rachtanapun C., Rachtanapun P. (2018). Biodegradable rice starch/carboxymethyl chitosan films with added propolis extract for potential use as active food packaging. Polymers.

[41] Khodabakhshi D., Eskandarinia A., Kefayat A., Rafienia M., Navid S., Karbasi S. (2019). In vitro and in vivo performance of a propolis-coated polyurethane wound dressing with high porosity and antibacterial efficacy. Colloids Surf. B Biointerfaces.

[42] Du P., Chen X., Chen Y., Li J., Lu Y., Li X. (2023). In vivo and in vitro studies of a propolis-enriched silk fibroin-gelatin composite nanofiber wound dressing. Heliyon.

[43] Eskandarinia A., Kefayat A., Gharakhloo M., Agheb M., Khodabakhshi D., Khorshidi M. (2020). A propolis enriched polyurethane-hyaluronic acid nanofibrous wound dressing with remarkable antibacterial and wound healing activities. Int. J. Biol. Macromol..

[44] Shao W., Wu J., Wang S., Huang M., Liu X., Zhang R. (2017). Construction of silver sulfadiazine loaded chitosan composite sponges as potential wound dressings. Carbohydr. Polym..

[45] Aramwit P., Muangman P., Namviriyachote N., Srichana T. (2010). In vitro evaluation of the antimicrobial effectiveness and moisture binding properties of wound dressings. Int. J. Mol. Sci..

[46] Loureiro K.C., Barbosa T.C., Nery M., Chaud M.V., da Silva C.F., Andrade L.N. (2020). Antibacterial activity of chitosan/collagen membranes containing red propolis extract. Die Pharmazie-An International Journal of Pharmaceutical Sciences.

[47] Seidel V., Peyfoon E., Watson D.G., Fearnley J. (2008). Comparative study of the antibacterial activity of propolis from different geographical and climatic zones. Phytother Res..

[48] Kalogeropoulos N., Konteles S.J., Troullidou E., Mourtzinos I., Karathanos V.T. (2009). Chemical composition, antioxidant activity and antimicrobial properties of propolis extracts from Greece and Cyprus. Food Chem..

[49] Drago L., Mombelli B., Vecchi Ed, Tocalli M.F.L., Gismondo M. (2000). In vitro antimicrobial activity of propolis dry extract. J. Chemother..

[50] Popova M., Reyes M., Le Conte Y., Bankova V. (2014). Propolis chemical composition and honeybee resistance against Varroa destructor. Nat. Prod. Res..

[51] Ota C., Unterkircher C., Fantinato V., Shimizu M. (2001). Antifungal activity of propolis on different species of Candida. Mycoses.

[52] Ramos A., Miranda Jd (2007). Propolis: a review of its anti-inflammatory and healing actions. J. Venom. Anim. Toxins Incl. Trop. Dis..

[53] Mj Y., Gh G. (2007).

[54] Abdulkhani A., Hosseinzadeh J., Ashori A., Esmaeeli H. (2017). Evaluation of the antibacterial activity of cellulose nanofibers/polylactic acid composites coated with ethanolic extract of propolis. Polym. Compos..

[55] Zeighampour F., Alihosseini F., Morshed M., Rahimi A.A. (2018). Comparison of prolonged antibacterial activity and release profile of propolis‐incorporated PVA nanofibrous mat, microfibrous mat, and film. J. Appl. Polym. Sci..

[56] Veiga R.S., De Mendonça S., Mendes P.B., Paulino N., Mimica M.J., Lagareiro Netto A.A., Lira I.S., López B.C., Negrão V., Marcucci M.C. (2017 Apr 1). Artepillin C and phenolic compounds responsible for antimicrobial and antioxidant activity of green propolis and Baccharis dracunculifolia DC. J. Appl. Microbiol..

[57] Gu B.K., Park S.J., Kim M.S., Kang C.M., Kim J.I., Kim C.H. (2013 Aug 14). Fabrication of sonicated chitosan nanofiber mat with enlarged porosity for use as hemostatic materials. Carbohydr. Polym..

[58] Chen Z.G., Wang P.W., Wei B., Mo X.M., Cui F.Z. (2010 Feb 1). Electrospun collagen–chitosan nanofiber: a biomimetic extracellular matrix for endothelial cell and smooth muscle cell. Acta Biomater..

[59] Mengistu Lemma S., Bossard F., Rinaudo M. (2016 Oct 26). Preparation of pure and stable chitosan nanofibers by electrospinning in the presence of poly (ethylene oxide). Int. J. Mol. Sci..

[60] Kashi T.S., Kermanshahi R.K., Erfan M., Dastjerdi E.V., Rezaei Y., Tabatabaei F.S. (2011). Evaluating the in-vitro antibacterial effect of Iranian propolis on oral microorganisms. Iran. J. Pharm. Res. (IJPR): IJPR.

[61] Uzel A., Sorkun K., Onçağ O., Cogŭlu D., Gençay O., Salih B. (2005). Chemical compositions and antimicrobial activities of four different Anatolian propolis samples. Microbiol. Res..

[62] Eskandarinia A., Kefayat A., Agheb M., Rafienia M., Amini Baghbadorani M., Navid S., Ebrahimpour K., Khodabakhshi D., Ghahremani F. (2020 Feb 20). A novel bilayer wound dressing composed of a dense polyurethane/propolis membrane and a biodegradable polycaprolactone/gelatin nanofibrous scaffold. Sci. Rep..

